# Reinforcement versus Fluidization in Cytoskeletal Mechanoresponsiveness

**DOI:** 10.1371/journal.pone.0005486

**Published:** 2009-05-08

**Authors:** Ramaswamy Krishnan, Chan Young Park, Yu-Chun Lin, Jere Mead, Richard T. Jaspers, Xavier Trepat, Guillaume Lenormand, Dhananjay Tambe, Alexander V. Smolensky, Andrew H. Knoll, James P. Butler, Jeffrey J. Fredberg

**Affiliations:** 1 Program in Molecular and Integrative Physiological Sciences, Harvard School of Public Health, Boston, Massachusetts, United States of America; 2 Research Institute MOVE, Faculty of Human Movement Sciences, VU University, Amsterdam, The Netherlands; 3 Unitat de Biofisica i Bioenginyeria, Universitat de Barcelona – IBEC, Barcelona, Spain; 4 Botanical Museum, Harvard University, Cambridge, Massachusetts, United States of America; Kings College London, United Kingdom

## Abstract

Every adherent eukaryotic cell exerts appreciable traction forces upon its substrate. Moreover, every resident cell within the heart, great vessels, bladder, gut or lung routinely experiences large periodic stretches. As an acute response to such stretches the cytoskeleton can stiffen, increase traction forces and reinforce, as reported by some, or can soften and fluidize, as reported more recently by our laboratory, but in any given circumstance it remains unknown which response might prevail or why. Using a novel nanotechnology, we show here that in loading conditions expected in most physiological circumstances the localized reinforcement response fails to scale up to the level of homogeneous cell stretch; fluidization trumps reinforcement. Whereas the reinforcement response is known to be mediated by upstream mechanosensing and downstream signaling, results presented here show the fluidization response to be altogether novel: it is a direct physical effect of mechanical force acting upon a structural lattice that is soft and fragile. Cytoskeletal softness and fragility, we argue, is consistent with early evolutionary adaptations of the eukaryotic cell to material properties of a soft inert microenvironment.

## Introduction

In the cell's repertoire of mechanical responses to imposed stretch – mechanoresponsiveness – the newly discovered existence of cytoskeletal fluidization[1] demonstrates that the cell can deploy not just one strategy, as previously believed, but two. The better known strategy is reinforcement.[2]–[4] Reinforcement causes rapid actin polymerization and increased focal adhesion assembly, resulting in increases in cytoskeletal stiffness and traction forces.[5]–[9] But in any adherent cell resident in an organ that stretches all the time, such as heart, lung, and gut, reinforcement-induced cell stiffening, if left unopposed, would progressively impede organ stretch and thus could become a self-defeating strategy. To maintain homeostasis, therefore, an opposing compensatory mechanism might become a biological necessity; Walter B. Cannon, originator of the concept of homeostasis, said in his book *Wisdom of the Body,* “when a factor is known which can shift a homeostatic state in one direction it is reasonable to look for a factor or factors having an opposing effect.”[10] Fluidization is now seen as being reinforcement's opposite, and is exemplified by prompt decreases of CSK stiffness and increases in macromolecular mobility.[1] In response to stretch, therefore, the cell might either reinforce, a bracing-type of physiological response, or fluidize, a stress-relieving physiological response. But are these opposing factors at work all the time, or at least in some circumstances, might one factor prevail over the other?

To answer these questions, here we used a novel approach that combines cell stretch with traction force microscopy.[3], [11]–[20] Compared with previous approaches, the experimental methods used here are more precise, entirely quantitative, and much simpler. Because it maps in space and in time the traction stress response to a well-defined imposed stretch, we call this method Cell Mapping Rheometry (CMR). Using CMR, we found that that the localized reinforcement response [2]–[4] fails to scale up to the level of the whole cell undergoing repetitive homogeneous cell stretch. Rather than stiffening, solidifying, and increasing traction forces above prestretch baseline values, as would be predicted from a reinforcement response, in most physiological circumstances the human airway smooth muscle cell promptly softens, fluidizes, and decreases traction force, with subsequent slow recoveries that approach but never exceed baseline values. In the remainder of this report we will refer to the former constellation of responses simply as reinforcement and the latter as fluidization.

## Results

### Cell Mapping Rheometry

**Figure 1:**
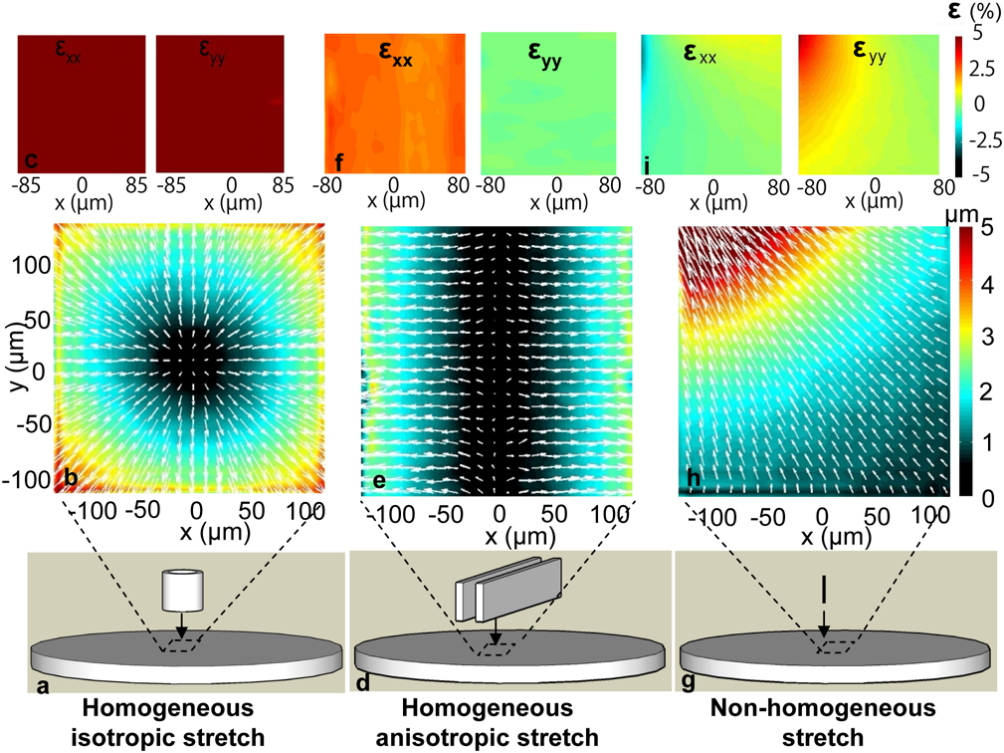
Cell Mapping Rheometry (CMR). Localized vector displacements in the gel are indicated by arrows, and their magnitude by color. a–c, Indentation of the gel with an annular punch indenter imposes a homogeneous isotropic biaxial stretch within the central region of the indenter. d–f, When the gel is indented with two parallel plates, the corresponding stretch field in the central gel region is homogeneous, anisotropic and uniaxial. g–i, When the gel is indented with a microneedle, a non-homogeneous stretch is imposed.

We plated the isolated human airway smooth muscle cell on a gel substrate and then applied stretch using a punch-indentation system (Figure 1). When an annular punch indents the gel, the region in the gel center bulges and its surface undergoes a strain that is biaxial and isotropic (Figure 1a–c). When parallel plates indent the gel, however, the strain field is uniaxial (Figure 1d–f). Depending upon the shape of the indenter, therefore, the cell adherent upon the gel surface can be subjected to a homogeneous stretch that is either biaxial and isotropic in the plane or uniaxial and anisotropic in the plane. Non-homogeneous fields of stretch can be prescribed as well (Figure 1g–i). If the punch is then lifted the gel recoils elastically and the cell will have undergone one cycle of transient stretch-unstretch.

### Cell traction forces decrease following a single homogeneous biaxial stretch

Tractions are the local physical forces that an adherent cell exerts upon its substrate, expressed as force per unit area (stress). We begin by posing a simple but important question, how do cell traction forces develop in space and time in response to a rapid transient isotropic biaxial cell stretch? One might reason that cell traction is similar to a first order strain energy derivative and cell stiffness is a second order derivative, but while this statement is true for passive materials it is not true for active biological materials wherein molecular motors and active polymerization responses can generate active stresses and traction forces that can be uncoupled from strain energy derivatives.

**Figure 2:**
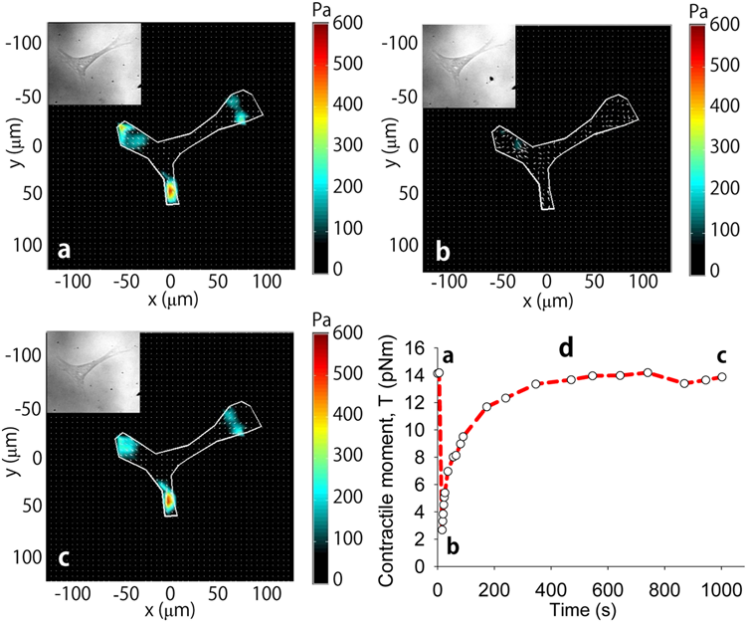
CMR measurements for a representative cell. a, Traction map before cell stretch. b, Traction map measured immediately after an imposed homogeneous biaxial stretch of a 4 second stretch-unstretch maneuver with a peak strain amplitude of 10%. The cell tractions are markedly ablated. c, Traction map measured at 1000 seconds following stress cessation. Tractions have largely recovered to the baseline pre-stretch value measured in (a). d, The traction field can be used to compute the contractile moment, T, corresponding to an equivalent force dipole.[14] At the earliest measurable time point following stretch (b), the contractile moment was significantly reduced to 20% of its baseline value (a) followed by a slow recovery (c).

**Figure 3:**
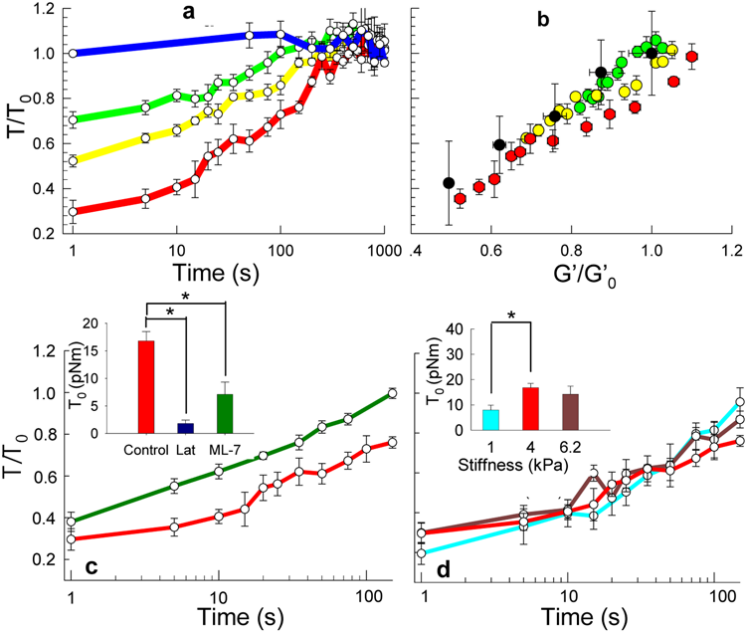
Traction dynamics following a homogeneous biaxial isotropic stretch. a, Tractions as represented by the contractile moment T relative to the unstretched baseline value T_0_ versus time, after stretch cessation. The greater was the applied stretch, the greater were the reductions in cell traction, and the faster were the recoveries. Peak strains of: 0 (blue; n = 9), 2.5 (green; n = 12), 5.0 (yellow; n = 11) and 10.0% (red; n = 14). b, When traction data from (a) are plotted not versus time, but rather versus the instantaneous value of the cell stiffness (G′), all data collapse.[15] Following a transient cell stretch, cell stiffness (x axis) and cell tractions (y axis) evolved in concert. Similar strong associations between stiffness and tractions have been measured previously[15] in response to graded concentrations of relaxing or contracting agonists, but exclusively under static steady-state conditions (black). c, Dynamic traction measurements in HASM cells treated with ML-7 (dark green; n = 13) or Latrunculin (dark blue; n = 13). Pharmacologically treated cells were found to exert significantly smaller tractions at baseline compared to untreated cells, often to levels below the measurable range. Observable traction responses to a 10% transient stretch relative to its unstretched baseline value T_0_ following stress cessation in ML-7 treated cells (n = 5) were similar in quality but markedly different in magnitude. d, Dynamic traction measurements in cells plated on soft (cyan; n = 7), intermediate (red; n = 14) and stiff (brown; n = 10) substrates (Young's moduli of 1,4, or 6.2 kPa). Despite differences in baseline pre-stretch tractions (inset, * p<0.05), in response to a 10% transient stretch, normalized traction changes were strikingly similar within the three stiffness groups.

After completion of a transient stretch-unstretch maneuver of 4 second duration (Figure S1a), the traction field indicated a dramatic and prompt decrease followed by slow recovery (Figure 2; Movie S1). Pre-stretch values of traction and projected cell area varied extensively between cells and approximated a log-normal distribution (data not shown). Nonetheless, for graded stretch magnitudes within the physiological range (2.5, 5 and 10% isotropic biaxial strains, respectively, with 0% strain corresponding to a time-control), resulting changes in traction were consistent between cells (Figure 3a). Moreover, as the magnitude of the stretch was increased, the prompt ablation of traction became progressively greater (p<0.005, two-tailed unpaired Student's *t* test). No changes in focal adhesion area were noted, however (Figure S5). Control samples with no applied stretch showed no changes of traction. The slow recovery also varied in a load-dependent manner with the largest stretch magnitude showing the fastest recovery.

### The fluidization response is robust

To assess the robustness of these responses, we pretreated cells with drugs whose effects on the cytoskeleton (CSK) have been well documented. Inhibition of myosin light chain kinase with ML7 or depolymerization of F-actin with Latrunculin-A reduced the pre-stretch tractions to levels far below those observed in untreated cells (Figure 3c, inset) and often to levels below the measurable range. Nonetheless, observable traction responses to a transient stretch (10% strain amplitude) were similar in quality but markedly different in magnitude (Figure 3c).

To assess further the generality of these responses, we plated the isolated HASM cell on substrates of different stiffness. We used CMR with soft, intermediate, or stiff substrates (Young's moduli of 1, 4, or 6.2 kPa, respectively) and characterized corresponding cell tractions and their changes. As reported previously in cells that are not subjected to stretch[15], [21], cells on substrates with progressively larger stiffness produced progressively larger static tractions (Figure 3d, inset; p<0.05, two-tailed unpaired Student's *t* test). Despite these static differences, dynamic traction responses within these three stiffness groups were similar (Figure 3d). Taken together, these findings suggest that under static conditions matrix rigidity acts as a tactile set-point to regulate cell traction forces, but dynamic responses to stretch are governed by mechanisms that appear to be invariant with regard to changes of substrate stiffness. Indeed, when we measured cell stiffness using Optical Magnetic Twisting Cytometry (OMTC)[1] and plotted changes of cell stiffness versus those of traction at the same time points and under identical experimental conditions, we found that changes in cell stiffness mirrored changes in cell traction in almost perfect synchrony and, remarkably, all data collapsed along a unifying linear relationship (Figure 3b). We have shown previously that cell stiffnesses and cell tractions vary in direct proportion[15], [22], but those earlier studies were restricted to steady-state conditions only. Our new data now establish that during dynamic maneuvers, and at timescales as short as 1 second, strong and inseparable relationships persist between cell traction and cytoskeletal fluidization.[1]

### The fluidization response does not depend upon stretch isotropy

The fluidization response stands in contrast to the reinforcement response associated with local cell stretch applied through an attached microbead or microneedle, in which case local stiffness and force are seen to increase and to do so on the time scale of seconds.[2]–[4] We questioned whether these paradoxical responses (fluidization versus reinforcement) might be reconciled by differences in cell responses to isotropic versus anisotropic stretch and the more complex state of intracellular mechanical stress in the latter case. To address this question we subjected each cell to a transient deformation that departed markedly from biaxial isotropic stretch and instead more closely approximated uniaxial cell stretch (Figure 1d–f). These experiments demonstrated a fluidization response closely similar to that observed during isotropic biaxial stretch (Figure S2).

### Localized cell stretch causes reinforcement, but homogeneous cell stretch does not

**Figure 4:**
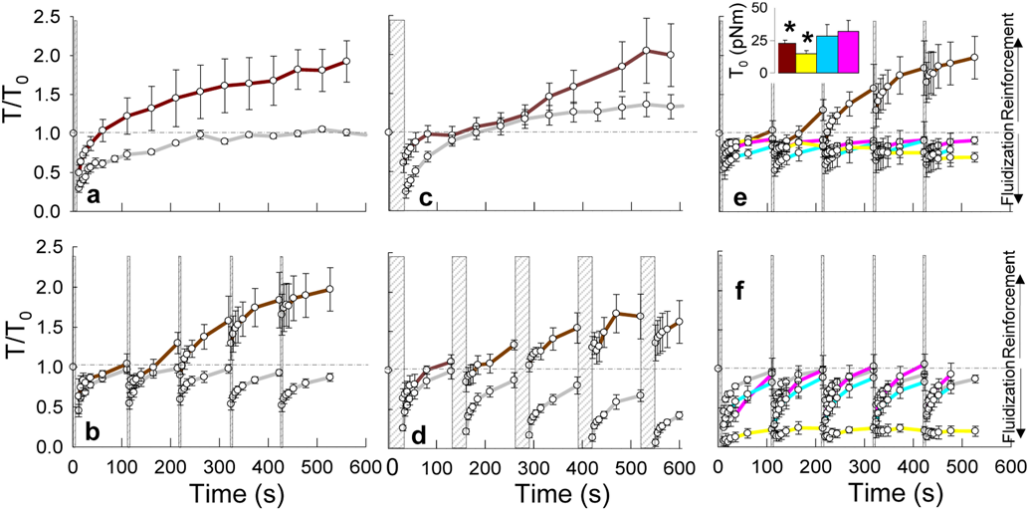
Homogeneous stretch induces fluidization; non-homogeneous stretch induces reinforcement. Traction as represented by the contractile moment T relative to the unstretched baseline value T_0_ versus time, after stretch cessation. a, In response to both homogeneous biaxial stretch (gray; 10% strain magnitude, duration = 4 sec, Table S1) and non-homogeneous stretch (brown; strain magnitude as in Figure 1 i, duration = 4 sec), we observed prompt cytoskeletal fluidization. The subsequent recoveries, however, differed dramatically. While tractions after non-homogeneous stretch crossed and then exceeded baseline value by as much as 75% (p = 0.017) at 600s, traction recovery after homogeneous stretch did not (p>0.05). b, In response to a time series of repetitive transient stretches (Figure S1 b), non-homogeneous stretch (red) exhibited reinforcement while homogeneous biaxial stretches (gray; 10% strain magnitude) did not; the tractions after every homogeneous stretch cycle became progressively smaller and smaller. c,d In response to transient stretches of a longer time duration (duration = 30 sec, Figure S1 c,d), qualitatively similar results were obtained. e, When cells were pretreated with 50nM phenylarsine oxide (PAO) (yellow), 10mM EGTA (pink) or 25 μM Gadolinium Chloride (blue) and subjected to a time series of repetitive non-homogeneous stretches (Figure S1 b), no reinforcement was observed. f, Alternatively, when treated cells were subjected to a time series of repetitive homogeneous stretches, the traction force recovery following each stretch cycle was markedly ablated only in the case of PAO treatment.

We then questioned whether reinforcement might alternatively be a response that is peculiar to stretch non-homogeneity. To explore this possibility we used a microneedle punch to induce a single transient non-homogeneous cell deformation (Figure 1g–i) much as did Munevar et al.[3] Both in the case of homogeneous biaxial stretch and nonhomogeneous stretch, we observed prompt cytoskeletal fluidization responses that were not different from each other (Figure 4a); the fluidization response did not depend upon stretch homogeneity. The subsequent recoveries, however, differed dramatically. Traction force recovery after non-homogeneous stretch crossed and then exceeded baseline value by as much as 92% (p = 0.04) at ∼600 s (Movie S2), which is a response consistent with reinforcement, while traction force recovery after homogeneous stretch did not exceed baseline (p>0.05; Figure 4a).

To assess further the generality of these responses, we subjected the cell to a transient stretch of longer duration (30 seconds, Figure S1c). Whereas traction recovery following a homogeneous biaxial stretch of 4 seconds duration never exceeded the prestretch baseline value, traction recovery following a homogeneous stretch of 30 second duration exceeded baseline by about 35% at 600 s (p = 0.08; Figure 4c), which is a response consistent with reinforcement. This reinforcement response was ablated, however, when homogeneous biaxial transient stretches were applied repetitively in a series (Figure S1b,d). Indeed, with each successive load cycle the traction force became progressively smaller (Figure 4b,d, Table S1). By contrast, when we used the microneedle to apply a comparable time series of repetitive non-homogeneous transient stretches, upon load removal we observed in every case a prompt fluidization response followed by what appeared to be reinforcement (Figure 4b,d, Table S1). To confirm further that this response corresponded to a reinforcement response, we pretreated cells with the tyrosine phosphatase inhibitor phenylarsine oxide (PAO).[2] When treated cells were subjected to a series of non-homogeneous stretches (Figure S1b), no reinforcement response was observed (Figure 4e). Alternatively, when treated cells were subjected to a series of homogenous biaxial stretches, the traction force recovery was highly sensitive to PAO but the prompt fluidization response was not. Next, we assessed the role of calcium in these responses. When cells were pretreated with the extracellular calcium chelating agent EGTA[23] or the stretch activated ion channel inhibitor Gadolinium Chloride[4] and subjected to a series of non-homogeneous stretch, the reinforcement response was ablated (Figure 4e). Alternatively, when treated cells were subjected to repeated homogenous stretch, tractions responses were largely unaffected (Figure 4f). Moreover, the time course of the calcium imaging suggested that the prompt fluidization responses could not be mediated by stretch-induced calcium signaling (Movie S3, Movie S4, Figure S6).

### The fluidization response is not restricted to the HASM cell

Although results reported here were limited to the HASM cell, similar experiments on bladder smooth muscle cells, human umbilical vein endothelial cells, and osteocytes yielded comparable results (data not shown).

## Discussion

### Physiological implications

Taken together, results reported here indicate that in response to repetitive load transients the reinforcement response in the HASM cell is peculiar to non-homogeneous cell deformations, as would occur during repetitive bead pulling or needle poking, but fails to scale up to the case of repetitive homogeneous cell stretch, whether isotropic or anisotropic. The reinforcement response and the fluidization response differ in sign, and this difference suggests either that reinforcement is not triggered by homogeneous cell stretch, or that reinforcement is somehow overwhelmed or blocked by the fluidization response. As regards the important issue of mechano-protection, the reinforcement response vis-à-vis the fluidization response both seem logical protective strategies – either brace for the storm or go with the flow. Nonetheless, in loading conditions that would be expected in most physiological circumstances the reinforcement response in the HASM cell was suppressed and the fluidization response prevailed. Given the complexity of signaling cascades that are triggered during reinforcement[2], [4], [6], [9], [24], [25], it seems unlikely that reinforcement might be an artifact of nonphysiologic loading conditions associated with microbead pulling or microneedle poking. As such, failure of the reinforcement response to scale up to the case of repetitive homogeneous cell stretches, as would be expected in ordinary physiological circumstances, is perplexing. This failure leads to the suggestion that the reinforcement response might serve some physiologic function other than mechano-protection, but whether reinforcement might be a response to cellular micro-injury that is not triggered by homogeneous cell stretch, for example, or might serve mainly to facilitate cellular adhesion or motility, is unclear.

### Mechanism

Transient stretch causes prompt detachment of the motor protein myosin from actin and a profound reduction in the myosin duty cycle[20], [26], [27]. Transient stretch also causes transient decreases in F-actin content.[23] Dynamics of these kinds by themselves are not sufficient to account for the fluidization response, however, because they fail to explain malleability of the cytoskeleton[27]–[30], its scale-free rheology[31], or its universality[1]. Moreover, myosin dynamics alone could hardly account for responses of opposite sign – fluidization versus reinforcement – that are observed in the cases of homogeneous versus nonhomogeneous cell stretch, respectively.

Protein dynamics in the complex functional system that define the cytoskeleton more generally are dominated by multiple weak interactions operating within a noisy thermal microenvironment but far from thermodynamic equilibrium[32], and we have suggested previously that these specific interactions among structural proteins are defined by energy wells that are just deep enough to avoid thermal insult but shallow enough to remain selectively responsive to physical forcing[1]. Similar physical effects have been recapitulated recently in purified actin solutions and interpreted using a non-equilibrium theory.[33], [34]

We do not speculate here on mechanisms that might account for the strange, unexpected, and altogether decisive role of nonhomogeneous versus homogeneous cell stretch. But as regards the latter, which is the more physiological case as regards mechanoresponsiveness, we suggest that the universality, the robustness, and, especially, the rapidity of the fluidization response, when taken together, lead to a remarkably simple physical picture: the cytoskeleton belongs to the special class of materials that are soft and fragile.[35] By using the word fragile in this context we mean to suggest that in loading conditions expected in most physiological circumstances, fluctuations in physical forces that are associated with routine cyclic organ stretch will induce within the stress bearing cytoskeletal lattice force-dependent molecular disassociations[16] of a most primitive kind – molecular dissociations that are prompt, transient, and non-specific. If so, then with each cell stretch physical forces could fluidize the cytoskeletal lattice. And since physical force is promiscuous in that it fails to respect the specific but weak molecular interactions that dominate the cytoskeletal lattice, then such an indiscriminant disruption of molecular interactions would explain how fluidization could trump reinforcement. Importantly, these events would be seen as being prompt and direct effects of physical force upon a wide range of weak molecular interactions, as opposed to being downstream events mediated by specific pathways of cell signaling.

This physical picture of the cytoskeleton as a fragile material does not at all rule out highly specific mediator-dependent cascades of signaling responses to cell stretch, including both reinforcement [2], [4], [6], [9], [24], [25] and resolidification[1]. It does, however, define strict physical limitations that were previously unappreciated and around which these signaling cascades must operate and may have evolved. In organs that are routinely subjected to cyclic stretch, such as great vessels, lung, and gut, these direct physical effects of cyclic stretch would be ever-present, inescapable, and dominant. In this connection, moreover, fluidization of inert versus living fragile matter in response to deformation demonstrates a unification of physical behaviors that is most striking but perhaps not coincidental. As developed in Text S1, there is good reason to question if this unification might reflect early events in eukaryotic cell evolution, including the earliest adaptations of the eukaryotic cell to material properties of a soft inert microenvironment.

## Methods

### Cell Mapping Rheometry (CMR)

Biaxial deformation was imposed on an elastic polyacrylamide substrate using a novel punch indentation system (Figure 1). The indenter was mounted to the microscope, coaxial to the objective lens. It was then lowered manually by a calibrated amount onto the underlying substrate in order to impose a predetermined strain. When the punch indents the gel, the region in the center bulges and in doing so its surface undergoes an approximately uniform biaxial or uniaxial strain depending on the shape of the indenter. Accordingly, the cell adherent upon that surface is subjected to a biaxial stretch that is isotropic in the plane (Figure 1a–c) or uniaxial (Figure 1d–f). If the punch is then lifted the gel recoils elastically and the cell will have undergone one cycle of transient stretch-unstretch. This deformation field can be applied and removed rapidly, and, by using indentations of defined depth, can create precisely controlled and highly repeatable cell strains that span the physiological range (Figure S3, Figure S4).

### Cell culture and pharmacological interventions

Human Airway Smooth Muscle (HASM) cells were isolated from tracheal muscle of lung transplant donors using a previously described method.[36] (cell source: University of Pennsylvania; no informed consent necessary for cell cultures as all donor identifiers were removed) The cells were cultured on plastic flasks in Ham's F-12 medium supplemented with 10% fetal bovine serum, 100 Uml^−1^ penicillin, 100 mgml^−1^ streptomycin, 200 mgml^−1^ amphotericin B, 12 mM NaOH, 1.7 mM CaCl_2_, 2 mM L-glutamine, and 25 mM HEPES. Once the cells in passage 4–6 reached confluence, they were serum deprived for 42 hours to arrest the cell growth cycle in the G1/G0 phases. The cells were then plated very sparsely (∼1,000cells/dish) in serum-free medium on type I collagen-coated (0.1 mg/ml) polyacrylamide gel dishes for 4–8 hours before being tested. The following pharmacological interventions were used to modulate the CSK filaments and baseline contractility: Latrunculin-A (disruption of F-actin via sequestration of actin monomers, 0.1 μM, incubation time = 20 min), ML7 (inhibition of myosin light chain kinase, 30 μM, incubation time = 10 min), Phenylarsine oxide (tyrosine phosphotase inhibitor, 50nM, incubation time = 15 min), EGTA (10mM, incubation time = 15 min) and Gadolinium Chloride (25 μM, incubation time = 15 min).

### Preparation of polyacrylamide gel substrates

Polyacrylamide gel substrates were prepared according to a previously described protocol.[13], [15] Briefly, 250 μl of an Acrylamide / Bis-acrylamide mixture dissolved in ultrapure water containing BIS-Acrylamide and Acrylamide (Bio-Rad, Hercules, CA) of different concentrations (1 kPa:5% Acrylamide and 0.03% Bis-acrylamide; 4 kPa:5% Acrylamide and 0.1% Bis-acrylamide;6.2 kPa:10% Acrylamide and 0.03% Bis-acrylamide), 0.6% of 0.2 μm diameter yellow fluorescent beads (Invitrogen, Eugene, OR), 0.5% of ammonia persulfate and 0.05% TEMED (Bio-Rad, Hercules, CA) was added to the center of each pre-treated dish [15] to yield gels with a final thickness of ∼700 μm. Following gelation, the surface was activated with 200 μl of a solution containing 1 mM Sulfosuccinimidyl-6-[4-azido-2-nitrophenylamino] hexanoate (Sulfo-SANPAH; Pierce, Rockford, IL), coated with 200 μl of type I Collagen solution (0.1 mg/ml; Inamed Biomaterials, Fremont, CA) and stored overnight at 4°C. On the following day, the gels were washed, hydrated with 2 ml of serum free media solution and stored in an incubator at 37°C and 5% CO_2_ until the day of the experiment.

### Experimental protocol

Single adherent HASM cells were subjected to the testing protocol illustrated in Figure S1. Phase contrast images of the cell and images of fluorescent microbeads embedded within the substrate and directly underneath the cell were taken at different time points during the no-load baseline period, before the onset of stretch, after stretch cessation and following cell detachment at the end of the experiment.

### Calculation of cell-tractions and contractile moment

Cell tractions were computed using constrained Fourier transform traction microscopy (FTTM).[14] Briefly, the displacement field was computed by comparing fluorescent microbead images obtained during the experiment with a reference image obtained at the end of the experiment subsequent to detaching the cell from its underlying substrate. The projected cell area was calculated based on a manual trace of the cell contour determined from a phase contrast image obtained at the start of the experiment. Both cell shape and cell area were found to vary only marginally during the experiment. From the displacement field we calculated the traction field, and from the traction field we computed a scalar measure of contractility called the contractile moment, T.[14] The contractile moment corresponds to the strength of an equivalent force dipole and provides thereby a scalar measure of the cell's contractile strength.

### Immunofluorescence staining

At the earliest time point following a 10% uniform transient stretch, cells were fixed with 5% formalin for 10 min, permeabilized for 5 min with 0.5% Triton X-100 (Sigma-Aldrich), blocked with a solution containing 5% goat serum and 1% BSA and subsequently stored overnight with a primary antibody directed against vinculin (V9131 monoclonal antivinculin antibody, Sigma-Aldrich). After three washes with PBS, cells were incubated at room temperature in the presence of a secondary antibody (Alexa Fluor 488 goat anti-mouse IgG antibody, Invitrogen). After two more washes, cells were counterstained with 1 μg/ml Hoechst 33342 (Sigma-Aldrich), mounted, and imaged with a 40× oil immersion lens. Stretched cells in each dish were compared with unstretched cells located outside the region of punch indentation. Focal adhesion area was computed by tracing the vinculin stained regions.

### Calcium imaging

The Fluo-4 NW Calcium Assay Kit (Invitrogen, Eugene, OR, USA) was used according to manufacturers' instructions. HASM cells were incubated at 37°C for 30 minutes in the dye loading solution dissolved in assay buffer (Component C, Invitrogen). Following incubation, the cells were washed twice with assay buffer, equilibrated at room temperature for an additional 30 minutes, and then stretched. Fluorescent measurements were recorded at several time points before and after stretch with a 20× objective lens.

## Supporting Information

Figure S1 Experimental protocol for dynamic cell traction measurements.
(0.02 MB PDF)

Figure S2 The fluidization response does not depend upon stretch isotropy. a–b, Anisotropic strains were imposed using two parallel plates. By modifying the ratio of plate separation to plate length, the major/minor strain ratio can be varied. For example, in a, the strain ratio = 30, which is nearly uniaxial whereas in b, the strain ratio = 2. c, In response to a transient stretch, the contractile moment T relative to the unstretched baseline value T0 promptly decreases followed by a slow recovery (red: 10% biaxial tensile strain plotted from Fig. 3a; black: ∼20% uniaxial strain, n = 6; green: anisotropic strain distribution from b, n = 5).
(0.85 MB PDF)

Figure S3 Applied biaxial stretch magnitudes are scalable within the physiological range and highly repeatable. a, Fluorescent bead marker positions embedded within a polyacrylamide gel (thickness = 700 Âμm) were obtained before and after a prescribed indentation with an annular punch with an inner and outer diameter of 2 mm and 3 mm, respectively. b–d, Gel displacement field in the central region (200×200 Âμm) corresponding to three different indentation depths of 150, 200 and 400 Âμm, respectively. The displacement field was calculated based on relative changes in embedded fluorescent bead marker positions. The arrows have been scaled by a factor of 4 for clarity. e, Despite different maximum displacement magnitudes in (b–d), the corresponding strain field is homogenous and uniform in the plane (b: Strain = 2.2%, r2 = 0.96; c: Strain = 5.6%, r2 = 0.99; d: Strain = 9.4%, r2 = 0.99). f, When forty closely spaced transient stretches (indentation depth = 400 Âμm) were applied consecutively, the applied strain was found to be highly reproducible between loading cycles. For all cycles, the displacement field was computed by comparing unloaded images at the end of a loading cycle with a loaded image taken at the start of the experiment.
(0.94 MB PDF)

Figure S4 Applied stretch can be tensile or compressive depending on indenter size. a–b, Finite element analysis of an elastic substrate (thickness = 2 mm; diameter = 20 mm; Young's modulus = 4 kPa; Poisson ratio = 0.48) indented to different depths (0.05 to 0.15 mm) with an annular punch of various cross-sectional diameters. The resulting radial strain field at the substrate surface is largely isotropic over the central region, scales with indentation depth and changes from a tensile to a compressive field for large diameter indenters. c, Displacement field measured experimentally in the central region (200×200 μm) for an annular indenter with inner and outer diameters of 8×9 mm, respectively. Resulting radial displacements are compressive (Arrows point inwards).
(3.10 MB PDF)

Figure S5 Focal adhesion area does not change immediately after a transient homogeneous stretch. a, Immunohistochemical staining for vinculin in an un-stretched cell. b, Immunohistochemical staining for vinculin immediately after a single transient biaxial stretch (Figure S1a). c, No notable change in focal adhesion area were observed at the earliest time point following a 10% biaxial stretch (when the ablation of the traction forces is the very greatest and the traction forces are the very smallest).
(0.70 MB PDF)

Figure S6 The time course of the fluidization response is not mediated by calcium signaling. Also see Figure 4 and Movies S3 and S4.
(0.02 MB PDF)

Table S1 Sample database for Figure 4
(0.02 MB PDF)

Text S1 Traction, glassy dynamics and the origin of eukaryotes
(0.02 MB PDF)

Movie S1 Traction dynamics following a single homogeneous biaxial stretch. The time lapse covers a period of 1000 seconds, with a single transient biaxial stretch applied between t = 5.6 seconds and t = 15.6 seconds.
(2.12 MB MOV)

Movie S2 Traction dynamics following a single non-homogeneous stretch. The time lapse covers a period of 600 seconds, with a single transient stretch applied between t = 2 seconds and t = 11 seconds.
(0.69 MB AVI)

Movie S3 Calcium imaging following a single transient homogeneous stretch (10% stretch magnitude) applied between t = 1 second and t = 11 seconds.
(6.14 MB AVI)

Movie S4 Calcium imaging following a single transient non-homogeneous stretch applied between t = 1 second and t = 11 seconds.
(0.85 MB AVI)
