# Supplementary figures and images for: Reinforcement versus Fluidization in Cytoskeletal Mechanoresponsiveness
Source: PLoS One. 2009 May 8;4(5):e5486. doi: 10.1371/journal.pone.0005486 (PMC2675060; doi:10.1371/journal.pone.0005486)

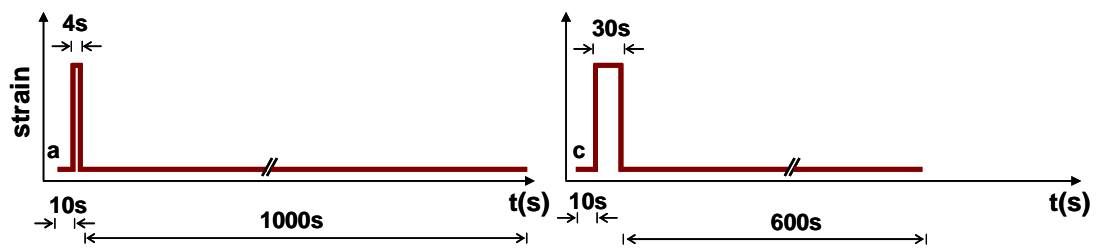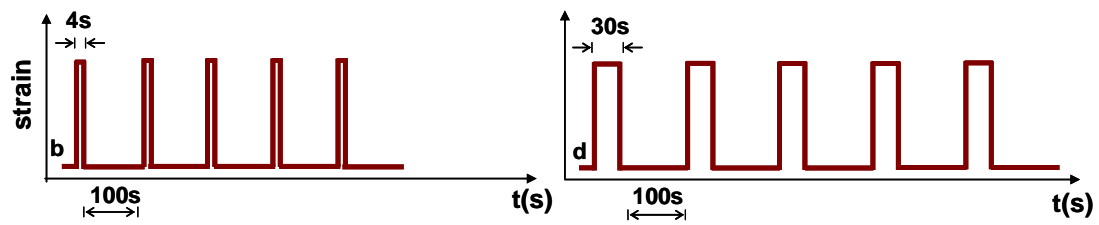

Supplement: Figure S1 — Experimental protocol for dynamic cell traction measurements. (0.02 MB PDF) [file pone.0005486.s001.pdf]

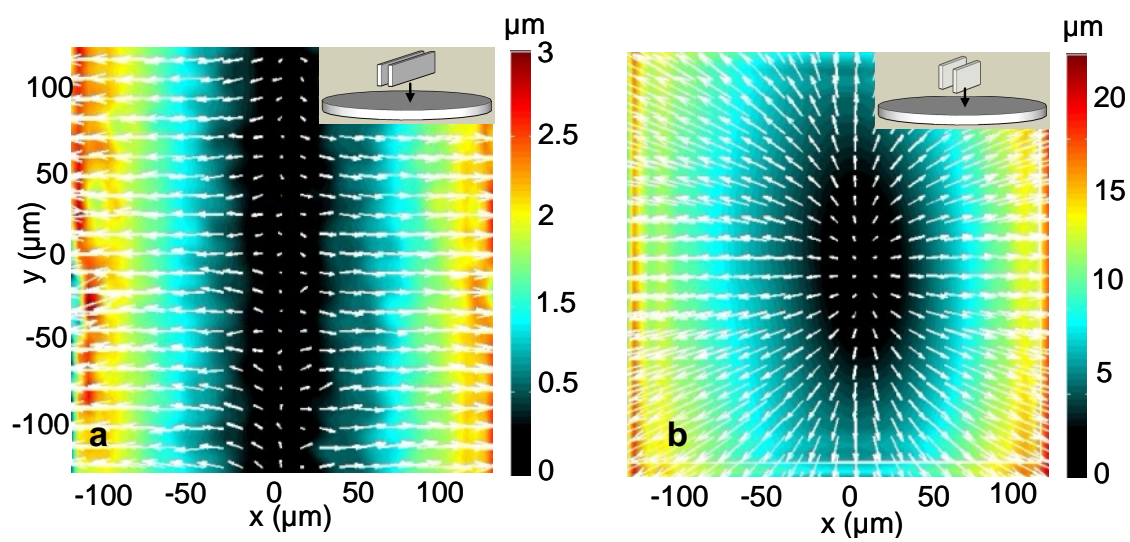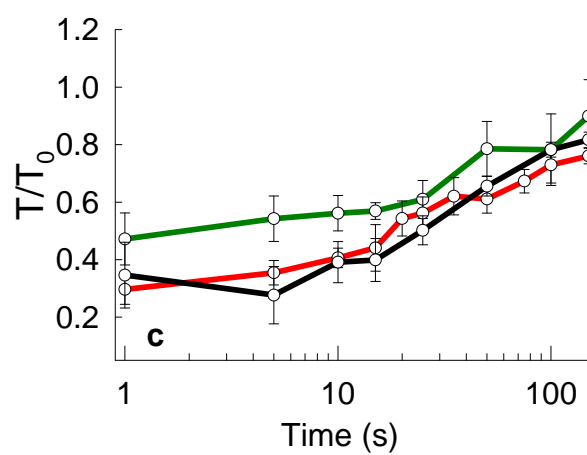

Supplement: Figure S2 — The fluidization response does not depend upon stretch isotropy. a–b, Anisotropic strains were imposed using two parallel plates. By modifying the ratio of plate separation to plate length, the major/minor strain ratio can be varied. For example, in a, the strain ratio = 30, which is nearly uniaxial whereas in b, the strain ratio = 2. c, In response to a transient stretch, the contractile moment T relative to the unstretched baseline value T0 promptly decreases followed by a slow recovery (red: 10% biaxial tensile strain plotted from Fig. 3a; black: ∼20% uniaxial strain, n = 6; green: anisotropic strain distribution from b, n = 5). (0.85 MB PDF) [file pone.0005486.s002.pdf]

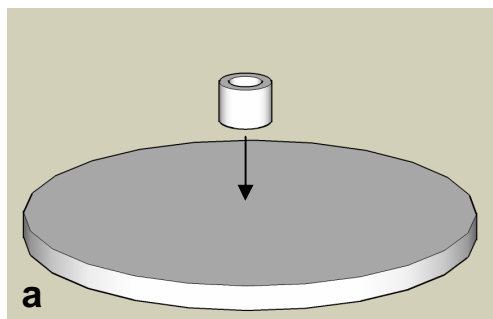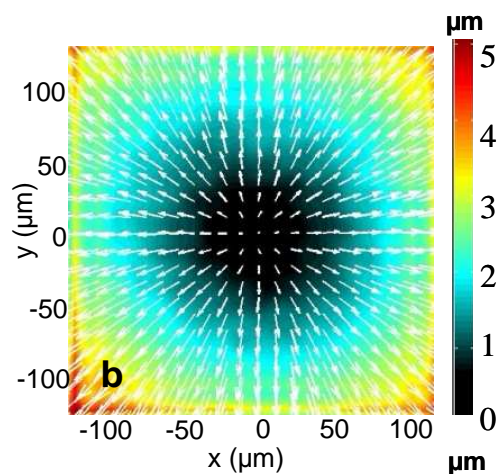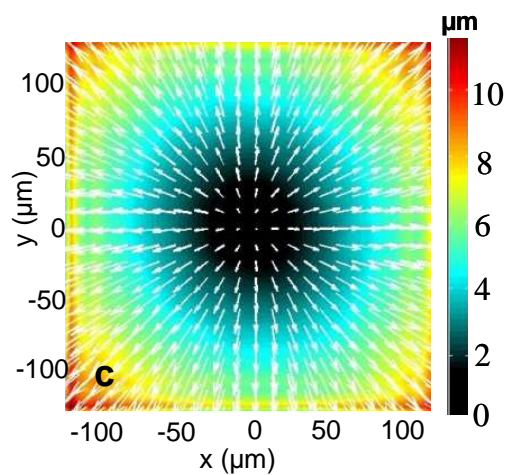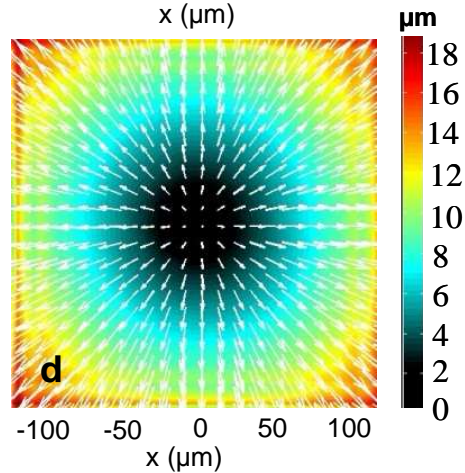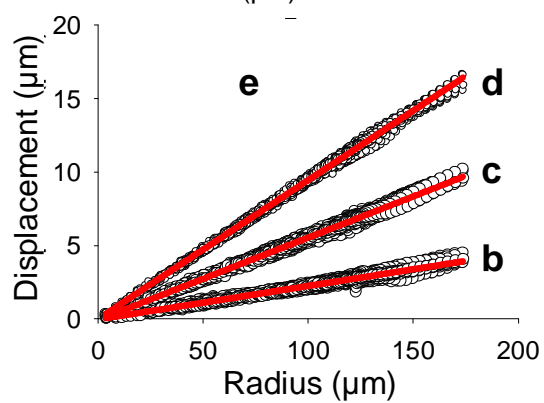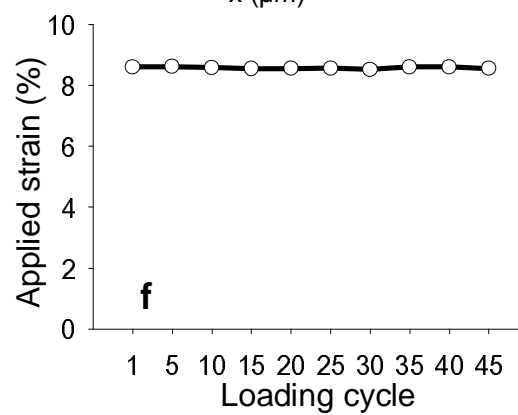

Supplement: Figure S3 — Applied biaxial stretch magnitudes are scalable within the physiological range and highly repeatable. a, Fluorescent bead marker positions embedded within a polyacrylamide gel (thickness = 700 Âμm) were obtained before and after a prescribed indentation with an annular punch with an inner and outer diameter of 2 mm and 3 mm, respectively. b–d, Gel displacement field in the central region (200×200 Âμm) corresponding to three different indentation depths of 150, 200 and 400 Âμm, respectively. The displacement field was calculated based on relative changes in embedded fluorescent bead marker positions. The arrows have been scaled by a factor of 4 for clarity. e, Despite different maximum displacement magnitudes in (b–d), the corresponding strain field is homogenous and uniform in the plane (b: Strain = 2.2%, r2 = 0.96; c: Strain = 5.6%, r2 = 0.99; d: Strain = 9.4%, r2 = 0.99). f, When forty closely spaced transient stretches (indentation depth = 400 Âμm) were applied consecutively, the applied strain was found to be highly reproducible between loading cycles. For all cycles, the displacement field was computed by comparing unloaded images at the end of a loading cycle with a loaded image taken at the start of the experiment. (0.94 MB PDF) [file pone.0005486.s003.pdf]

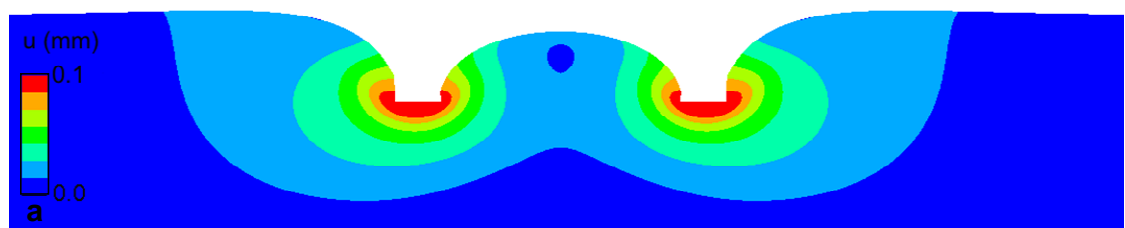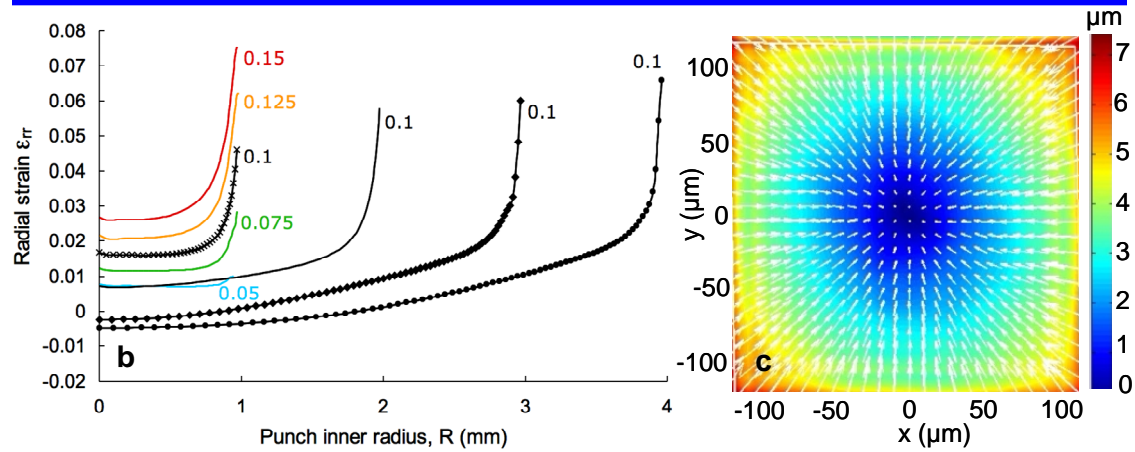

Supplement: Figure S4 — Applied stretch can be tensile or compressive depending on indenter size. a–b, Finite element analysis of an elastic substrate (thickness = 2 mm; diameter = 20 mm; Young's modulus = 4 kPa; Poisson ratio = 0.48) indented to different depths (0.05 to 0.15 mm) with an annular punch of various cross-sectional diameters. The resulting radial strain field at the substrate surface is largely isotropic over the central region, scales with indentation depth and changes from a tensile to a compressive field for large diameter indenters. c, Displacement field measured experimentally in the central region (200×200 μm) for an annular indenter with inner and outer diameters of 8×9 mm, respectively. Resulting radial displacements are compressive (Arrows point inwards). (3.10 MB PDF) [file pone.0005486.s004.pdf]

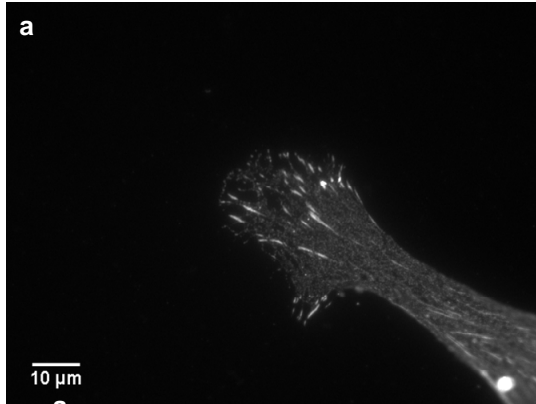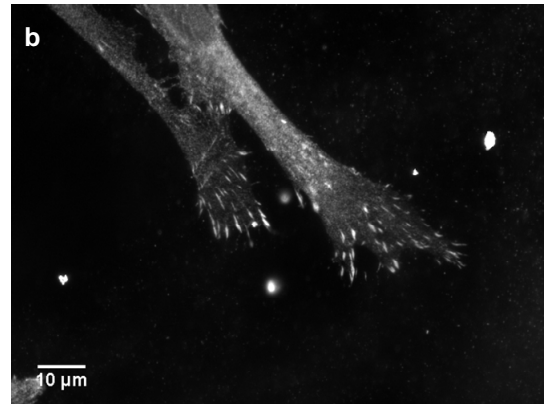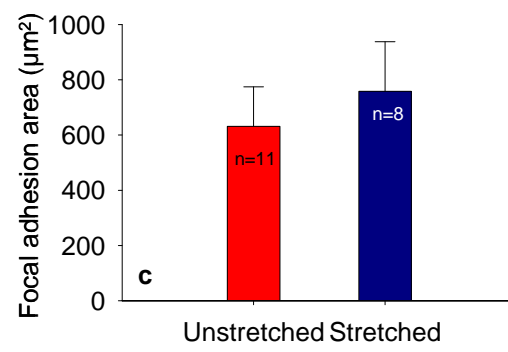

Supplement: Figure S5 — Focal adhesion area does not change immediately after a transient homogeneous stretch. a, Immunohistochemical staining for vinculin in an un-stretched cell. b, Immunohistochemical staining for vinculin immediately after a single transient biaxial stretch (Figure S1a). c, No notable change in focal adhesion area were observed at the earliest time point following a 10% biaxial stretch (when the ablation of the traction forces is the very greatest and the traction forces are the very smallest). (0.70 MB PDF) [file pone.0005486.s005.pdf]

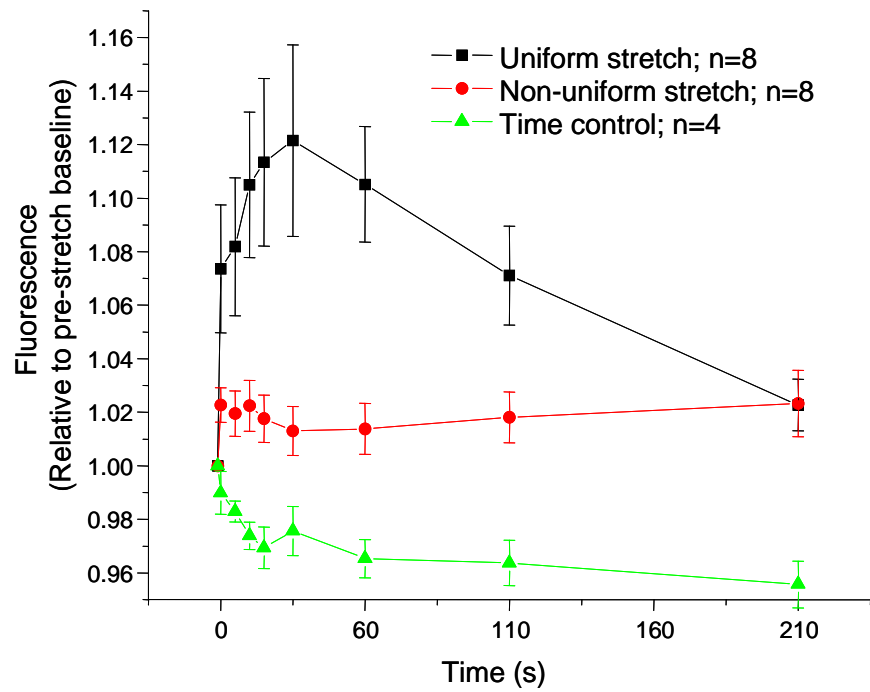

Supplement: Figure S6 — The time course of the fluidization response is not mediated by calcium signaling. Also see Figure 4 and Movies S3 and S4. (0.02 MB PDF) [file pone.0005486.s006.pdf]
