# Supplementary material for: Reinforcement versus Fluidization in Cytoskeletal Mechanoresponsiveness
Source: PLoS One. 2009 May 8;4(5):e5486. doi: 10.1371/journal.pone.0005486 (PMC2675060; doi:10.1371/journal.pone.0005486)
Supplement: Table S1 — Sample database for Figure 4 (0.02 MB PDF) [file pone.0005486.s007.pdf]

	Contractile moments at baseline, T_0 (pNm) average ±std. error	No. of cells (n)	Concentration (M) and incubation time (min)
HASM<br>Homogeneous,4s,single(gray)<br>Non-homogenous, 4s,single(brown)<br><br>Homogeneous,4s,multiple(gray)<br>Non-homogenous,4s,multiple(brown)<br><br>Homogeneous,30s,single(gray)<br>Non-homogenous,30s,single(brown)<br><br>Homogeneous,30s,multiple(gray)<br>Non-homogenous,30s,multiple(brown)	28.88±5.34*	90<br>12<br>5<br><br>5<br>4<br><br>5<br>9<br><br>5<br>5	-
HASM PAO<br>Homogeneous,4s,multiple(yellow)<br>Non-homogeneous,4s,multiple(yellow)	15.35±2.20*	24<br>4<br>6	50 nM; 15 min
HASM EGTA<br>Homogeneous,4s,multiple(blue)<br>Non-homogeneous,4s,multiple(blue)	29.22±11.23	14<br>4<br>5	10 mM; 15 min
HASM GdCl3<br>Homogeneous,4s,multiple(pink)<br>Non-homogeneous,4s,multiple(pink)	32.7±8.77	12<br>5<br>6	25 µM; 15 min

* $p < 0.05$
