# Supplementary material for: Reinforcement versus Fluidization in Cytoskeletal Mechanoresponsiveness
Source: PLoS One. 2009 May 8;4(5):e5486. doi: 10.1371/journal.pone.0005486 (PMC2675060; doi:10.1371/journal.pone.0005486)
Supplement: Text S1 — Traction, glassy dynamics and the origin of eukaryotes (0.02 MB PDF) [file pone.0005486.s008.pdf]

Text S1

Traction, glassy dynamics and the origin of eukaryotes

Ample evidence from the current study and previous publications [1-8] now confirms that glassy dynamics, including fluidization, are highly conserved across cell types, and implies that the cell closely regulates its traction stresses and material properties. How might these highly conserved behaviors have arisen from events occurring early in eukaryotic evolution?

Current phylogenies for the Eukarya do not identify any one clade as basal within the domain, but it is clear that the last common ancestor (LCA) of living eukaryotes was a motile heterotroph able to ingest particulate organic matter. [9,10] Indeed, the presence in the LCA of a mitochondrion acquired via endosymbiosis indicates that slow remodeling of cytoplasm made possible by glassy behavior [5,6] is primitive within the domain and may have arisen as early as the cytoskeleton itself. Bacteria and Archaea evolved more than three billion years ago, but stem group eukaryotes likely expanded only after the initial oxygenation of the atmosphere and surface ocean about 2.4 billion years ago. [11] Modern day prokaryotes have turgor pressure as large as 0.5-50 atmospheres, or about 10^5 -10^7 Pa, and exhibit effective elastic shear moduli in roughly the same range.[12] The eukaryote by contrast is characterized by elastic moduli that are smaller by three to four orders of magnitude.[3,7] Prokaryotes take up nutrients by diffusion alone [13] , whereas the loss of a stiff cell wall and development of a soft cytoskeleton allowed eukaryotes to move and capture particles using flexible cell modifications like pseudopodia to ingest particulates via phagocytosis [14,15] That is, the glassy behavior of eukaryotic cytoplasm may have facilitated eukaryotic radiation by

endowing early protists with functional capabilities not shared by the prokaryotes that dominated Earth's earliest ecosystems.

Measurements reported here suggest an additional environmental benefit of a cytoskeleton that is soft and glassy with material properties set by traction stresses. Is it a matter of mere happenstance, we wondered, that elastic properties and dynamics of eukaryotic cells are closely comparable to those of nutrient-rich microbial mats and detritus likely to have accumulated on the seafloor or lake bottom? For a long time in evolution such microbial mats must have represented a nutrient-rich but unexploited environmental niche. Mats and sediments comprising uncompacted soft matter would have an elastic shear modulus characteristic of other soft glassy matter, on the order of 10^3 Pa.[4,16] Had early eukaryotes been substantially less stiff than such sediments, and only able to develop tractions that are small compared with shear moduli of this surrounding media, invasion of these environments would have been mechanically impractical. But were the primitive eukaryote much stiffer than such sediments, then the cell tractions and cell deformations that drive invasion would become prohibitively costly with regard to metabolism. Although reasons for loss of the rigid prokaryotic cell wall remain obscure [13], once that catastrophic event transpired the early eukaryote could secure shape stability only through the agency of its internal cytoskeletal machinery, and we establish here that traction forces that the cell generates, and that would be required for invasion, are tuned to match shear moduli of the cell interior and the surrounding microenvironment. Accordingly, this new experimental evidence supports the point of view that mechanical properties of the soft inert microenvironment of early ancestors

may have exerted a selective pressure that dictated the magnitude of traction stresses and elastic moduli of modern day eukaryotes.

It is impossible to tell whether phagocytosis or movement through soft sediments came first, but both would have facilitated the evolution of primitive, heterotrophic eukaryotes. With this in mind, the striking analogy between mechanical properties of inert versus living soft matter might be not so much a matter of a curious coincidence as much as a matter of a selective pressure that acted to set internal scales of traction, stiffness, and friction for the primitive cytoskeleton, its molecular machinery, and was conserved in all eukaryotes that followed - in the nomenclature of Kirschner and Gerhart [17], a conserved core process. Although invasion and phagotrophy may have evolved in parallel and are logically synergistic, invasion places upon cell material properties and associated traction stresses firm bounds that are experimentally testable whereas phagotrophy does not.

References

1. Pratusevich VR, Seow CY, Ford LE (1995) Plasticity in canine airway smooth muscle. J Gen Physiol 105: 73-94.
2. Fredberg JJ, Inouye DS, Mijailovich SM, Butler JP (1999) Perturbed equilibrium of myosin binding in airway smooth muscle and its implications in bronchospasm. Am J Respir Crit Care Med 159: 959-967.
3. Fabry B, Maksym GN, Butler JP, Glogauer M, Navajas D, et al. (2001) Scaling the microrheology of living cells. Phys Rev Lett 87: 148102.
4. Fabry B, Fredberg JJ (2003) Remodeling of the airway smooth muscle cell: are we built of glass? Respir Physiol Neurobiol 137: 109-124.
5. Bursac P, Lenormand G, Fabry B, Oliver M, Weitz DA, et al. (2005) Cytoskeletal remodelling and slow dynamics in the living cell. Nat Mater 4: 557-571.
6. Lenormand G, Bursac P, Butler JP, Fredberg JJ (2007) Out-of-equilibrium dynamics in the cytoskeleton of the living cell. Phys Rev E Stat Nonlin Soft Matter Phys 76: 041901.
7. Hoffman BD, Massiera G, Van Citters KM, Crocker JC (2006) The consensus mechanics of cultured mammalian cells. Proc Natl Acad Sci U S A 103: 10259-10264.
8. Trepat X, Deng L, An S, Navajas D, Tschumperlin D, et al. (2007) Universal physical responses to stretch in the living cell. Nature 447: 592-595.
9. Cavalier-Smith T (2002) The phagotrophic origin of eukaryotes and phylogenetic classification of Protozoa. Int J Syst Evol Microbiol 52: 297-354.
10. Baldauf SL (2003) The deep roots of eukaryotes. Science 300: 1703-1706.
11. Knoll AH (2003) Life on a Young Planet: The First Three Billion Years of Evolution on Earth. Princeton: Princeton University Press.
12. Arnoldi M, Fritz M, Bauerlein E, Radmacher M, Sackmann E, et al. (2000) Bacterial turgor pressure can be measured by atomic force microscopy. Phys Rev E Stat Phys Plasmas Fluids Relat Interdiscip Topics 62: 1034-1044.
13. Smith JM, Szathmary E (1995) The Major Transitions in Evolution. Oxford: Oxford University Press.
14. Doolittle RF (1995) The origins and evolution of eukaryotic proteins. Philos Trans R Soc Lond B Biol Sci 349: 235-240.
15. Mitchison TJ (1995) Evolution of a dynamic cytoskeleton. Philos Trans R Soc Lond B Biol Sci 349: 299-304.
16. Sollich P (1998) Rheological constitutive equation for a model of soft glassy materials. Physical Review E 58: 738-759.
17. Kirschner MW, Gerhart JC (2005) The Plausibility of Life. New Haven and London: Yale University Press.
